# Anemia of Chronic Kidney Disease—A Narrative Review of Its Pathophysiology, Diagnosis, and Management

**DOI:** 10.3390/biomedicines12061191

**Published:** 2024-05-27

**Authors:** Krzysztof Badura, Jędrzej Janc, Joanna Wąsik, Szymon Gnitecki, Sylwia Skwira, Ewelina Młynarska, Jacek Rysz, Beata Franczyk

**Affiliations:** 1 Department of Nephrocardiology, Medical University of Lodz, Ul. Zeromskiego 113, 90-549 Lodz, Poland; 2 Department of Nephrology, Hypertension and Family Medicine, Medical University of Lodz, Ul. Zeromskiego 113, 90-549 Lodz, Poland

**Keywords:** anemia, chronic kidney disease, iron, erythropoietin, ferritin, hemoglobin

## Abstract

Anemia is one of the most common chronic kidney disease (CKD) complications. It negatively affects patients’ quality of life and clinical outcomes. The pathophysiology of anemia in CKD involves the interplay of various factors such as erythropoietin (EPO) deficiency, iron dysregulation, chronic inflammation, bone marrow dysfunction, and nutritional deficiencies. Despite recent advances in understanding this condition, anemia still remains a serious clinical challenge in population of patients with CKD. Several guidelines have been published with the aim to systematize the diagnostic approach and treatment of anemia; however, due to emerging data, many recommendations vary between publications. Recent studies indicate a potential of novel biomarkers to evaluate anemia and related conditions such as iron deficiency, which is often present in CKD patients. Our article aims to summarize the pathophysiology of anemia in CKD, as well as the diagnosis and management of this condition, including novel therapeutic approaches such as hypoxia-inducible factor-prolyl hydroxylase inhibitors (HIF-PHI). Understanding these complex subjects is crucial for a targeted approach to diagnose and treat patients with anemia in CKD effectively.

## 1. Introduction

Anemia is a common complication in chronic kidney disease (CKD) patients, with a multifactorial pathophysiology involving impaired erythropoiesis, decreased erythropoietin (EPO) production, and dysregulated iron metabolism [1].

Traditionally, anemia is defined based on serum hemoglobin level; serum transferrin saturation (TSAT), an indicator of circulating iron; and serum ferritin, an indicator of stored iron. The Kidney Disease: Improving Global Outcomes guidelines define anemia as Hb < 13 g/dL in males and <12 g/dL in females. In CKD, definition of iron deficiency varies between several guidelines. Thus, we aimed to evaluate differences in recommended thresholds and criteria to diagnose ID. Anemia prevalence rises notably as kidney function declines, affecting one in five individuals with moderate CKD (estimated glomerular filtration rate [eGFR] 30 to 59 mL/min/1.73 m^2^) [2].

The understanding of anemia in CKD has evolved significantly, with research shedding light on the interplay of factors such as EPO deficiency, iron and hepcidin dysregulation, chronic inflammation, bone marrow dysfunction, a reduced red cell life span, or vitamin B12 or folic acid deficiencies [3,4,5,6]. Anemia in CKD is not only a marker of disease severity but also a predictor of adverse outcomes, including cardiovascular events and mortality [7]. Moreover, anemia is associated with worse quality of life and increased hospitalization risk compared to patients without this condition. This means more healthcare resources are being used to treat CKD patients with anemia comorbidity, which increases healthcare costs [8].

As the landscape of anemia management in CKD continues to evolve, there is a growing recognition of the need for comprehensive, multifaceted approaches that address the diverse mechanisms involved in the development of anemia in this patient population [7]. Traditional management strategies for anemia in CKD have primarily focused on erythropoiesis-stimulating agents (ESAs) and iron supplementation, but novel therapeutic options such as HIF-prolyl hydroxylase inhibitors (HIF-PHIs) are emerging as promising interventions [3]. These novel agents target the hypoxia-inducible factor (HIF) pathway to stimulate endogenous EPO production and improve iron utilization [9]. In a narrative review, we provide an insight into emerging theories in pathophysiology as well as diagnostic challenges and novel therapeutic methods of CKD-related anemia.

## 2. Pathophysiology

### 2.1. Erythropoetin Dysregulation

EPO is a glycoprotein hormone that regulates the process of erythropoiesis by preventing erythroid progenitors from apoptosis. Erythropoiesis begins with hematopoietic stem cells (HSCs) lineage decision where HSCs differentiate to megakaryocytic-erythroid progenitors. Afterwards, megakaryocytic-erythroid progenitors enter erythroid lineage and differentiate to burst-forming unit-erythroid (BFU-E). The next stage of erythroid differentiation begins with colony-forming unit-erythroid (CFU-E) formation, that is, the first cell with the expression of EPO receptor (EPOR). CFU-E gives rise to proerythroblasts that subsequently evolve into basophilic erythroblasts. It should be noted that CFU-E, proerythroblasts, and basophilic erythroblasts are highly responsive to EPO due to EPOR presence and form the EPO-dependent stage of erythroid differentiation [10].

A relative lack of EPO is considered to be a well-known cause of renal anemia [11]. Patients suffering from CKD have inadequately low EPO levels with respect to the degree of anemia [3]. Kidneys are the main source of EPO, which is produced in the peritubular type I interstitial cells located in the cortex or in the outer layer of the renal medulla, between the basolateral membrane of the proximal tubules and peritubular capillaries [12]. The main stimulus for synthesis of EPO is a tissue hypoxia [13]. Hypoxia-inducible factor 1 (HIF-1) is a transcription factor that is responsible for the regulation of EPO formation by binding to the hypoxia response element on the EPO gene [14]. Low oxygenation levels subsequently result in diminished stability and transcriptional activity of hypoxia-inducible factors (HIFs) [13]. HIFs comprise α (HIF-1α, -2α, and -3α) and β subunits, where the β subunit is always present, while the α subunit is controlled by oxygen levels through prolyl hydroxylase (PHD) enzymes [15]. PHDs catalyze the hydroxylation of HIF-1α under oxygen-rich circumstances, yet under hypoxia, HIF-2α emerges as the principal driver in enhancing EPO synthesis in both the kidneys and liver [10,16].

Reduction in EPO production by the kidney may occur as kidney function declines [17]. Individuals with pathological conditions involving the kidneys are more prone to anemia development that is not adequately compensated by sufficient EPO production [1]. The absence of EPO leads to programmed apoptosis mediated by the Fas antigen [11]. Its severity grows rapidly once eGFR falls below 30 mL/min/1.73 m^2^ [3]. There are other factors in CKD that contribute to EPO dysregulation such as proinflammatory cytokines or the interfering role of hepcidin in RBC production [11]. Addressing this deficiency along with other contributing factors is essential for understanding complex interplay between EPO and anemia in CKD to implement targeted interventions for affected patients. 

### 2.2. Iron Dysregulation and Inflammation

#### 2.2.1. General Mechanisms of Iron Stores Disturbances

Iron is a key component for hemoglobin production and is essential for erythropoietic response to EPO [18]. Numerous functions of iron in organism explain several features that occur in iron deficient patients with CKD, such as reduced quality of life more frequent and longer hospitalizations [3,19]. Iron can be obtained from a wide variety of foods and is mainly released from internal storage or salvaged from aging erythrocytes, whereas it is lost during exfoliation of the intestinal epithelium and blood loss [18,20]. Patients with CKD often lose large amounts of iron through blood loss and uremia. Moreover, chronic inflammation associated with CKD promotes hepcidin production, an acute phase protein, which decrease iron absorption from intestine and iron release from macrophages [1,3,19].

#### 2.2.2. Chronic Inflammation

Several studies have demonstrated elevated levels of inflammatory biomarkers such as interleukin (IL)-1, IL-6, interferon (IFN)-γ, C-reactive protein (CRP), and tumor necrosis factor alpha (TNF-α) in the progression of CKD [21]. The presence of chronic inflammation in CKD patients is driven by a vast range of underlying factors, such as uremic toxins, arteriosclerosis, higher level of proinflammatory cytokines, and general activation of the immune system [22]. Chronic inflammation may contribute to the variability in Hb levels and hypo-responsiveness to ESA [22]. Inflammation affects erythropoiesis by the inhibition of hypoxia-induced EPO production in Hep3B cells [22]. Certain proinflammatory cytokines such as TNF-α and IL-1 may suppress erythroid progenitor cell proliferation through the indirect pathway by inhibiting EPO production [21]. Another mechanism is the modulation of iron metabolism, which appears to be the critical pathway by which inflammation drives anemia [21]. Inflammation is the factor of both absolute and functional iron deficiency, which impairs the ability to utilize the iron sequestered in the tissues, causing erythropoiesis to be impossible [23,24]. Elevated ferritin levels, diminished iron, and iron-binding capacity, as well as higher abundance of iron in the bone marrow, are the characteristic features of inflammation-associated anemia [22]. Iron metabolism is further regulated by hepcidin [23]. Control of hepcidin expression and production by inflammation occurs in reaction to liver iron levels, hypoxia, and anemia. This leads to either functional iron deficiency or increased ferritin and reduced transferrin production, diverting iron to the reticuloendothelial storage pool rather than supplying it to erythrocyte precursors [22]. This abnormal escalation of hepcidin levels in hyperinflammatory state becomes a primary element of refractory renal anemia [25]. 

#### 2.2.3. Hepcidin

Hepcidin is a crucial regulator of circulating iron absorption and has an important role in CKD-related anemia pathophysiology, where elevated levels of hepcidin are often observed [26]. It is mainly synthesized by hepatocytes localized in the region of portal veins that carry dietary iron and Kupffer cells. Smaller amounts of hepcidin are also formed and secreted by macrophages and adipocytes [27]. Its production is influenced by various factors like hypoxia, anemia, erythropoietin, transferrin saturation, and liver iron levels, which can all be altered in CKD patients [28]. In response to inflammation, hepcidin expression is regulated by cytokines and bacterial lipopolysaccharide, affecting iron distribution and utilization. Inflammatory-induced hepcidin production may contribute to erythropoietin resistance by impeding erythroid progenitor proliferation [29]. Hepcidin also facilitates iron storage by promoting the internalization and degradation of ferroportin, preventing the release of iron into circulation from enterocytes, macrophages, or other iron stores [30]. Moreover, it should be noted that eGFR decrease leads to hepcidin clearance decrease, as it is primarily eliminated through the kidneys [3]. The resulting imbalance between excessive hepcidin secretion and decreased elimination leads to impaired erythropoiesis that worsen anemia in CKD patients.

### 2.3. Bone Marrow Dysfunction

Bone marrow dysfunction is one of the investigated pathways that contributes to anemia in CKD. A study with the aim to compare the structure of bone marrow in healthy patients and in those with CKD has shown that 53.3% of patients with CKD had hypocellular bone marrow [31]. There is no clear explanation why CKD promotes bone marrow hypocellularity [32]. Emerging theories to clarify this phenomenon indicate that uremic toxins have a potential to inhibit bone marrow function, subsequently leading to in impaired erythropoiesis. The likelihood of developing bone marrow hypocellularity was reduced with an increase in megakaryocytes; however, this association is not fully explained [31,32]. 

### 2.4. Vitamin B, Folic Acid, and Hyperhomocysteinemia

Patients with CKD often suffer from micronutrient deficiencies [1,33]. Water-soluble vitamin B12 also called cobalamin plays an important role in maintaining homeostasis, cellular metabolism, antioxidation, and red blood cell synthesis [33,34]. It is supplied to the body mainly with food like liver, meat, seafood, or milk [33,34,35]. CKD is often accompanied by diabetes that may impair the absorption of this vitamin, whereas a demand for vitamin B12 may be increased [35]. In most of these patients, additional supplementation may be necessary [33]. Currently, there is no evidence of a direct relationship between cobalamin deficiency and CKD, but the influence of hyperhomocysteinemia induced by cobalamin deficiency might affect the progression of CKD [34,35]. In CKD, cobalamin metabolites are indicators of functional status, but most patients with CKD have vitamin B12 levels within the normal range [34,35]. Vitamin B12 supplementation is also recommended for patients receiving folic acid [33,34]. 

In patients with CKD, folic acid has been shown to lower homocysteine levels, which in turn might lower the risk of cardiovascular disease and improve health outcomes in this population [36]. Hyperhomocysteinemia supports reactive oxygen and nitrogen radicle formation, which reduces the bioavailability of nitric oxide. As a result, collagen accumulates, which affects vascular remodeling and progressive changes in the vascular system, which causes damage to the microcirculation in the glomeruli and may also cause glomerular sclerosis and acute kidney damage [34,35,37]. As a consequence, greater degree of proliferation of vascular smooth muscle cells subsequently leading to hardening of are associated with podocyte function disruption that increases progression of renal fibrosis [35]. Folic acid deficiency can result in anemia and other health issues, underscoring the importance of ensuring sufficient folic acid intake for overall health and to prevent complications [38]. It is essential to consider the potential impact of folic acid on oxidative stress, cytotoxicity, and fibrogenic changes in kidney epithelial cells, as higher concentrations of folic acid may have adverse effects on renal function [39]. Despite the relevance of folic acid in managing anemia, its role in CKD requires further investigation to optimize treatment strategies and improve patient outcomes.

### 2.5. Bone Morphogenetic Protein 6 (BMP-6)

The role of bone morphogenetic protein (BMP) signaling in CKD-related anemia is complex and has been the subject of extensive research. Existing evidence indicates that BMP signaling plays a crucial role in regulating hepcidin, whereas the absence of BMP signaling has been shown to induce iron overload [40,41]. These findings emphasize the significance of BMP-6 iron imbalance management and show its potential as a targeted treatment for conditions linked to iron metabolism and anemia. Further research is needed for proper understanding of its role in CKD-related anemia.

### 2.6. Fibroblast Growth Factor 23 (FGF-23)

Fibroblast growth factor 23 (FGF-23) is a hormone produced by osteocytes and osteoblasts [42]. There is a positive correlation between FGF-23 and phosphorus levels, whereas a negative correlation with hemoglobin levels has been shown, suggesting a role in the development of anemia in CKD patients [43]. Studies indicated that FGF-23 suppresses erythropoiesis in CKD through a Klotho-independent mechanism; this suggests that FGF-23 could directly contribute to the development of anemia [44]. Furthermore, high levels of FGF-23 in CKD patients have been linked to reduced activation of neutrophils, which could affect the body’s immune response and potentially worsen anemia [45]. Although intravenous iron can decrease FGF-23 levels, it has not been determined whether lowering it will improve treatment outcomes [46,47,48]. More research is needed to determine the specific mechanisms through which FGF-23 influences erythropoiesis and contributes to anemia in CKD patients.

### 2.7. Other Factors

There are other factors such as several drugs that may contribute to the development of anemia in patients with CKD. Interestingly, widely used angiotensin convertase inhibitors (ACEI) may inhibit the synthesis of EPO and lead to further Hb decrease. This phenomenon can be explained by increased oxygenation in the periurethral area and a decrease in resistance in the efferent arterioles, accompanied by an increase in the concentration of IGF-1 in the serum [1,49]. Pharmacotherapy is also a cause of anemia in patients after kidney transplantation. In this group, anemia is estimated to occur in approximately 40% of patients due to immunosuppressive treatment. The antiproliferative effect on bone marrow has been demonstrated in drugs such as azathioprine, mycolate mofetil, and mycolate sodium [1].

**Figure 1:**
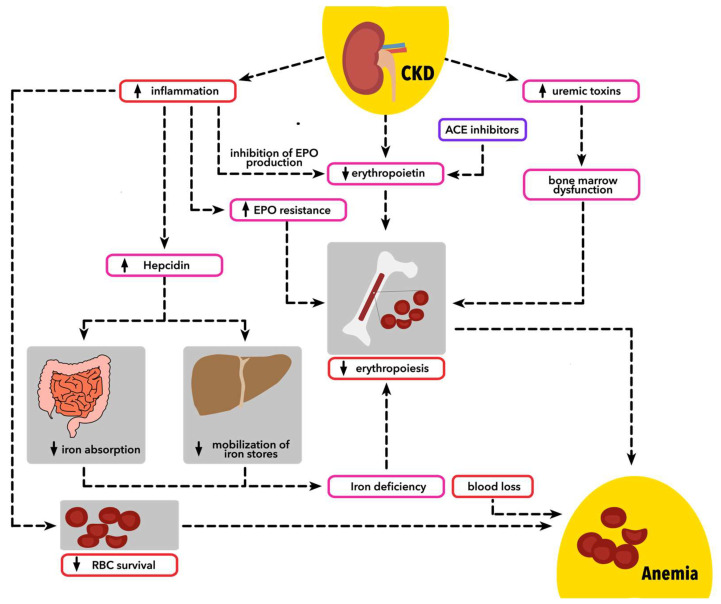
Pathophysiology of anemia in chronic kidney disease. CKD—chronic kidney disease; EPO—erythropoietin; ACE—angiotensin converting enzyme; RBC—red blood cells.

Bleeding is claimed to be an important cause of mortality among patients with renal failure; however, it can modify the clinical course of anemia of CKD. A recent meta-analysis comprising more than 7 million individuals from 22 studies has shown the rate of gastrointestinal bleeding (GIB) of 2.2% in patients with CKD. Factors associated with GIB are advanced patient age, diabetes, liver cirrhosis, and history of ulcers [50]. Underlying mechanisms of increased risk of bleeding in individuals with impaired renal functions are mainly associated with impaired platelet function and altered blood rheology [51]. Moreover, patients with CKD are at risk of morbidities requiring anticoagulation, such as venous thromboembolism and atrial fibrillation [52]. In non-dialyzed patient’s intrinsic platelet function remains impaired with coexisting disturbances of platelet with vessel wall interaction. Decreased expression of GpIb receptor in endothelium with reduced affinity to von Willebrand factor leads to impaired platelet adhesion. Moreover, improper function of GpIIb/IIIa has been observed, which is associated with impaired platelet aggregation. Increased nitric oxide and prostaglandin I2 levels commonly observed in uremic state are associated with the inhibition of platelet activation and vasodilation. Finally, decreased function of cyclooxygenases leads to low levels of thromboxane A2. Interestingly, anemia may also constitute an underlying cause of excessive bleeding in patients with CKD. It has been shown, that decreased hematocrit is associated with intravascular platelet flow disturbance, where platelets are within the central part of vascular lumen, that hinders platelet–wall interaction [51,53]. General pathophysiology of anemia in CKD has been shown on Figure 1. 

## 3. Diagnosis

### 3.1. Screening for Anemia

Anemia may be an initial sign of ongoing pathological condition in any individual. Thus, complete blood count is claimed to be a part of general assessment of patents’ health. According to the KDIGO guideline for anemia in CKD, patients without anemia should have Hb measurement following dictated frequency depending on CKD stage and renal replacement therapy (RRT) status and in every clinical condition requiring complete blood cell measurement. Hb measurement frequency in patients suffering from CKD without anemia is presented on Table 1 [54]. These guidelines released in 2012 seem to be relevant according to the latest data from cross-sectional studies assessing the prevalence of anemia among patients with CKD. Data from the Japan Chronic Kidney Disease Database (J-CKD-DB) comprising 31,082 patients with eGFR of 5–60 mL/min/1.73 m^2^ indicate that 40.1% of patients with CKD stage G4 had coexisting anemia, whereas in patients with stage G5, the prevalence of anemia was 60.3%. It should be noted that stages of CKD were significantly associated with anemia prevalence, which may confirm the reasonability of increased testing frequency in individuals with more advanced CKD. The authors of the J-CKD-DB analysis have shown the following diagnoses of CKD stages: G3b (OR 2.32, 95% CI 2.09–2.58), G4 (OR 5.50, 95% CI 4.80–6.31), and G5 (OR 9.75, 95% CI 8.13–11.7) [55]. An analysis based on cross-sectional data from the National Health and Nutrition Examination Survey (NHANES) III indicates that the prevalence of anemia in CKD individuals was 7.6% and increased with stage of CKD from 8.4% at stage G1 to 53.4% at stage G5 [56]. 

### 3.2. Diagnosis of Anemia

According to KDIGO guidelines, anemia should be diagnosed in adults and children >15 years with CKD when Hb concentration is <13 g/dl in males and <12 g/dL in females [54]. Interestingly, Hb cutoff values to diagnose anemia vary on several guidelines and recommendations. Renal association recommends that all patients with Hb < 11 g/dL and/or anemia symptoms should be investigated for possible causes of anemia and potential treatment [57]. On the other hand, recent World Health Organization (WHO) guidelines on Hb cutoffs define anemia in individuals aged between 15 and 65 years old as <12 g/dL for non-pregnant woman and <13 g/dL for men [58]. During pregnancy, cutoffs vary on trimester. This definition has been modified by KDOQI, which suggests diagnosis of anemia in patients with Hb < 13.5 g/dL in men and Hb < 12 g/dL in women [59].

The WHO definition of anemia adapted in KDIGO guidelines is based on large-scale multi-ethnic population datasets [54]. Consistent with recent WHO guidelines, established Hb cutoffs were mostly based on clinical symptoms and/or functional impairment; however, this approach was not possible in all age groups and pregnancy trimesters. For this definition, the fifth percentile has been selected with the aim to increase sensitivity in populations at risk of anemia [58]. As well as the WHO, KDOQI guidelines based on the NHANES dataset define cutoffs as follows: <12 g/dL in females and <13.5 in males [59]. These cutoff values are consistent with latest ERBP statement. The ERBP Working Group claims that the WHO definition could be useful for epidemiological purposes; however, it may miss a number of anemic European patients. The rationale for higher cutoff claims that in Caucasian men, a Hb setpoint is 1–2 g/dL higher when compared to African Americans. Thus, higher cutoff value for males has been proposed. Moreover, it should be noted that these cutoffs are suggested only for the European population [60]. 

### 3.3. Evaluation of Anemia

All patients with anemia and CKD should undergo further laboratory tests in initial evaluation to identify underlying causes of anemia. KDIGO guidelines as well as the Renal Association Clinical Practice Guideline on Anemia of Chronic Kidney Disease recommend following tests [54,57]:

- complete blood count (CBC), including mean corpuscular hemoglobin (MCH), mean corpuscular volume (MCV), mean corpuscular hemoglobin concentration (MCHC), white blood cell count, and platelet count.
- absolute reticulocyte count.

Moreover, a high prevalence of iron deficiency (ID) in non-dialysis patients with anemia has been observed. ID was present in 68.6% of females and 53.8% of males with CKD and anemia [61]. Thus, iron status assessment seems to be crucial to determine the cause and to consider proper treatment of anemia among CKD patients. According to iron status, it is recommended to assess [54]

- TSAT.
- serum ferritin.

The renal association guideline recommends assessing the percentage of hypochromic red blood cells (%HRC) and reticulocyte Hb count (CHr) (or equivalent tests) prior to TSAT and ferritin; however, TSAT and ferritin should be assessed when %HRC and CHr are not available [57]. As ferritin is accepted to be an inflammation biomarker, additional C-reactive protein measurements should be considered to exclude ongoing inflammation and prevent from false positive measurements of ferritin [54,57,62]. 

In addition to the measurements above, the KDIGO as well as the Renal Association recommend assessing vitamin B_12_ and foliate levels in the differential diagnosis of anemia; however, vitamin B_12_ and/or foliate deficiency in CKD patients seem to be less common, especially when compared to ID [54,57]. 

### 3.4. Iron Status Assessment

To define ID in patients with anemia and CKD, it is crucial to perform TSAT and ferritin measurements. International expert opinion on ID across chronic inflammatory conditions suggest that in patients with CKD, absolute ID should be defined as TSAT ≤ 20% and serum ferritin concentration ≤ 100 ng/mL (100 mcg/L) [63]. Some authors recommend different cutoffs among non-dialysis, peritoneal dialysis (PD), and hemodialysis patients (HD). Commonly recommended cutoffs to diagnose absolute ID in non-dialysis individuals and in patients undergoing PD are ferritin ≤100 ng/mL (100 mcg/L) and TSAT ≤ 20%, whereas in patients undergoing HD, ferritin concentration <200 ng/mL (200 mcg/L) and TSAT ≤ 20% is crucial to diagnose absolute ID [59,63,64]. It should be noted that cutoffs to diagnose absolute ID vary according to different guidelines due to highly inconclusive data about optimal values of ferritin and TSAT in decreased renal function (see the sections below). Key differences in cutoffs of recommended measurements to evaluate anemia in CKD patients are presented in Table 2. 

Another type of ID observed in patients with CKD, especially during erythropoiesis-stimulating agent (ESA) treatment, is functional deficiency where total body iron stores are adequate; however, release of iron storages is not rapid enough, subsequently causing insufficient iron availability for increased erythropoiesis. In these cases, iron status assessment reveals TSAT ≤ 20%, whereas serum ferritin is often increased [64].

#### 3.4.1. Serum Ferritin

Ferritin is the main iron storage protein with an ability to store about 4000–5000 iron atoms by each molecule [65,66,67]. In most of the tissues, it is present in the cytosol; however, recent data indicate the presence of mitochondrial and nuclear forms. Interestingly, apart from iron storage, ferritin can play important role in various physiological processes. It has been confirmed that mitochondrial forms of ferritin may protect against oxidative stress and further apoptosis, whereas nuclear forms protect DNA from damage due to the Fenton reaction [67,68,69,70]. Serum ferritin can carry iron without the ability to load labile extracellular iron and results from macrophages, hepatocytes, and Kupfer cells [67,71]. As already mentioned, serum ferritin concentration is an applied biomarker to assess iron content in the body; however, it can be also useful in the diagnosis of inflammatory disease and prognosis estimation in cancers [67]. 

It should be noted that levels of serum ferritin are different in patients with CKD when compared to normal renal function individuals [64]. The underlying cause of different cutoffs is CKD-associated hyperferritinemia that may occur in patients with decreased renal function. A review of Kalantar-Zadeh et al. [72] indicates the important role of inflammation and liver dysfunction in serum ferritin increase among CKD patients. Despite increased ferritin levels, iron overload is rarely observed. It has been suggested that ferritin levels above 2000 ng/mL are indicative for iron overload; however, most of the iron overload cases have been observed before ESA implementation. Excessive blood transfusions to manage anemia are claimed to be the main reason for hemosiderosis in this group of patients. Thus, it is claimed that serum ferritin levels <2000 ng/mL are rather associated with inflammation or liver disease than with iron overload [72]. In patients with normal renal function, serum ferritin concentration <30 ng/mL indicates ID, whereas in patients with CKD, other cutoff values have been proposed due to the studies indicating that ferritin >30 ng/mL does not indicate sufficient iron storage [54,59,60,64]. Several studies have been conducted to determine cutoff values of serum ferritin to determine ID among CKD patients. A study conducted by Kalantar-Zadeh et al. [73] with the aim to determine clinical application of ferritin and TSAT has shown that in anemic patients with CKD, serum ferritin <200 ng/mL is 100% specific for ID diagnosis. Results of the study of Domrongkitchaiporn et al. [74] indicate that in patients undergoing PD serum ferritin <100 ng/mL was also associated with 100% specificity, whereas serum ferritin <200 ng/mL had 83.3% specificity. Both cutoffs were associated with relatively low sensitivity—13.3% and 20%, respectively [74]. Interestingly, KDIGO guidelines emphasize that patients undergoing HD will only have normal bone marrow iron stores when their serum ferritin is ≥300 ng/mL (≥300 mcg/L), which calls previously presented cutoff into question [54].

It should be noted that relatively high serum ferritin cutoff of <500 ng/mL to diagnose ID presented in KDIGO guidelines may increase the sensitivity and increase the number of patients qualified for iron therapy. Although more patients may benefit from iron supplementation in Hb increase, the safety of an iron therapy remains questionable. A study conducted by Canavese et al. [75] has shown that there is a high probability of iron overload, even in patients with serum ferritin below 500 ng/mL, whereas a ferritin level >340 ng/mL has the highest performance to diagnose iron overload with area under the receiver operating characteristic curve (AUROC) of 0.716. Recent studies report lower optimal cutoff for ferritin to diagnose iron overload. Results of the study conducted by Rostoker et al. [76] indicate that optimal ferritin cutoff to diagnose iron overload of 162 ng/mL. Highly inconclusive data emphasize the need for further studies to assess optimal cutoffs for ID diagnosis and revise the indications for iron supplementation regarding non-dialyzed and dialyzed patients. 

#### 3.4.2. Transferrin Saturation (TSAT)

The primary role of transferrin is to transport iron in redox-inactive form around the human body. It is mostly synthetized in hepatocytes; however, other cells such Sertoli, ependymal, and cancer cells represent the expression of transferrin. Factors such as temperature, pH, and chelator and ionic concentrations influence the binding release of transported iron [77,78]. TSAT represents calculated total serum iron/total iron binding capacity (TIBC) × 100, whereas TIBC is calculated as transferrin × 1389. In the non-CKD population, TSAT ≤ 15% indicates ID; however, in CKD, higher cutoff levels are recommended [54,59,60,64]. Proposed cutoffs range between TSAT < 20% and TSAT < 30% [54,59,60,61]. In CKD, TIBC decreases from an average of 360 mcg/dL to 240 mcg/dL, which is associated with progressively decreasing renal function. Moreover, it has been suggested that a lower limit of serum iron of 45 mcg/dL remains not applicable to CKD patients. In CKD, individuals with serum iron above 45 mcg/dL may also suffer from ID. Therefore, a cutoff of 60 mcg/dL seems to be more applicable, especially in patients with stages G4 and G5 of CKD. To achieve serum iron above 60 mcg/dL in stage 4 and 5, Besarab et al. [79] suggests maintaining TSAT at least within the range of 22–26%; however, some patients might still have functional ID. Thus, it is questionable if fixed TSAT cutoff of 20% is adequate for 4 and 5 CKD stages [79].

According to KDIGO guidelines, the proposed TSAT cutoff of <30% to introduce iron supplementation is significantly higher when compared to the KDOQI, Renal Association guidelines, and ERBP opinion [54,57,59,60]. Moreover, no cutoff distinction between ND and HD/PD patients has been proposed [54]. ERBP opinion emphasizes that the median TSAT in the European population is 23%, that is, below the proposed cutoff by KDIGO and may lead to unnecessary iron supplementation in non-iron-depleted individuals [54,60]. It should be noted that the KDOQI and Renal Association Guidelines recommend lower TSAT cutoff to diagnose iron deficiency, whereas ERBP recommends different cutoffs for ND and HD/PD patients [54,57]. A TSAT cutoff of <30% to introduce iron supplementation has been proposed in the ERBP opinion as applicable to patients receiving ESA if Hb increase and/or ESA dose reduction is desired [60]. Analysis of over 142,000 patients undergoing HD revealed that significant elevation of Hb was associated with TSAT increase up to 20%. Moreover, among individuals receiving ESA therapy, Hb was significantly lower among patients with TSAT < 20%. ESA hypo-responsiveness decreased with an increase in TSAT up to 40%, whereas TSAT > 40% was associated with a decrease in Hb [80]. Therefore, the TSAT cutoff value of 20% seems reasonable. On the other hand, the study of Kuragano et al. [81] indicates a need for further evaluation of higher TSAT cutoff. This study with the aim to assess the role of serum ferritin and TSAT as predictors of cerebrovascular and cardiovascular disease and death among patients on HD revealed that patients with low TSAT (<20%) and increased ferritin (≥100 ng/mL) had significantly higher risk of cerebrovascular and cardiovascular disease (hazard ratio (HR) 4.45 95% CI 1.94–10.2; *p* < 0.001) and death (HR 5.80 95% CI 2.27–14.84; *p* < 0.001). Interestingly, patients with TSAT within 20–30% had significantly lower risk of developing cardiovascular and cerebrovascular diseases, whereas TSAT ≥ 30% was further associated with lower risk of death [81]. There is a need for further studies to evaluate appropriate TSAT and ferritin cutoffs in a reference to non-dialyzed, dialyzed, and/or ESA recipients. 

#### 3.4.3. Reticulocyte Hemoglobin Count (CHr)

Another method to diagnose functional ID is to assess CHr, which directly reflects the effectiveness of Hb synthesis in bone marrow precursors and can be used to measure iron availability. It is derived from the measurement of cell volume and hemoglobin content in reticulocyte [82]. When compared to TSAT, CHr has less factors influencing its values, indicating lower risk of potentially inaccurate measurements. Fishbane et al. [83] have compared CHr to ferritin and TSAT as a guide for iron treatment in hemodialysis patients. The results of the study indicate that CHr has significantly less variation than TSAT and ferritin (coefficient variation: 3.4% vs. 43.6% and 39.5% for ferritin and TSAT, respectively) [83]. Moreover, reticulocytes remain for approximately 24–48 h and can inform iron sufficiency immediately. Ogawa et al. [84] summarized studies aiming to determine cutoffs to diagnose ID, sensitivity, and specificity of CHr with comparison to TSAT and ferritin. Cutoffs to diagnose ID ranged from <29 pg up to <32.2 pg [84,85,86,87,88]. Sensitivity of CHr was 47–100%, whereas sensitivity of TSAT was 35–58.5 (for TSAT cutoff <19%) [84,85,86,87,88]. When compared to ferritin, sensitivities of ferritin were 19.6% (for cutoff <50 ng/mL) and 65% (for cutoff <100 ng/mL) [84,85,86,87,88]. To compare biomarkers, the authors of these studies have calculated the area under the receiver operating characteristic curve (AUROC). In hemodialyzed patients, an AUROC of CHr ranged from 0.60 up to 0.95 and has been shown to be significantly higher when compared to ferritin and TSAT (0.53–0.633 and 0.56–0.758, respectively) [84,86,87,88,89]. Thus, CHr seems to be a more accurate marker for the diagnosis and treatment monitoring of iron deficiency among CKD patients. A cutoff to diagnose iron deficiency should be established approximately between 29 pg and 32 pg. The highest AUROC for ID confirmation has been calculated for 32 pg cutoff; however, small groups and variable baseline characteristics limits the inference from these studies [84]. On the other hand, a study of Dinh et al. [90] has shown a high performance of CHr to predict ID anemia in end-stage renal disease patients with AUROC up to 0.979. Specificity and sensitivity for CHr at the cutoff of 29.4 pg were 99.3 and 98.3%, respectively [90]. Moreover, CHr has an ability to discriminate ID anemia and non-ID anemia patients (AUROC 0.748, 95% CI 0.656–0.840). An optimal cutoff to discriminate these two groups is 31.5 pg [90]. 

#### 3.4.4. Percentage of Hypochromic Red Cells (%HRC)

Hypochromic red cells are produced when ID occurs and comprises hypochromic reticulocytes, subsequently forming erythrocytes. Percentage of hypochromic red cells (%HRC) directly informs Hb synthesis; however, it does not provide a real-time iron sufficiency estimation due to mature erythrocytes that are measured in this method. An observational study of Kee et al. [89] has shown that in patients receiving hemodialysis, iron concentration and CHr in patients with %HRC ≤ 3.1% were significantly higher than in the group with %HRC > 3.1% at baseline (69.0 vs. 57.0 for iron and 32.6 vs. 31.8 for CHr, *p* < 0.05). Moreover, the incidence of anemia after 1 month follow-up was higher in patients with %HRC > 3.1% than in those with %HRC ≤ 3.1% (70.4% vs. 6.9%, *p* < 0.001), and also a negative correlation between Hb increase after one month and %HRC has been confirmed. Performance of %HRC as an ID predictor was significantly higher than CHr (AUROC 0.88, 95% CI 0.82–0.93 vs. 0.60, 95% CI 0.51–0.68) and TSAT (AUROC 0.56, 95% CI 0.47–0.64). The authors of this study have revealed that a cutoff value of %HRC to predict anemia development was 4.3%, with a sensitivity of 67.74% and specificity of 97.5% [89]. 

### 3.5. Other and Novel Biomarkers in Anemia of Chronic Kidney Disease Evaluation

#### 3.5.1. Soluble Transferrin Receptor (sTfR)

Soluble transferrin receptor (sTfR) is an extracellular portion of membranous glycoprotein contributing to iron transport through the cellular membrane and is present on most of the cells, especially in requiring high amounts of iron [91,92]. Several studies have confirmed the utility of sTfR to assess iron status, indicating comparable ability in diagnosing iron deficiency anemia to ferritin; however, it is not influenced by inflammation [92]. In patients with CKD, sTfR correlated with Hb; however, there was no association between sTfR and the presence of iron deficiency in patients with positive response to ESA [93]. Moreover, a study conducted by Fusaro et al. [94] has shown that sTfR positively correlated with CHr in patients undergoing HD; however, there was no significant association between sTfR, ferritin, and TSAT. Interestingly, a prognostic role of sTfR has been confirmed, where high sTfR levels positively correlated with all-cause mortality regardless ID and anemia presence (hazard ratio (HR) 1.77 95% CI 1.05–2.98). Moreover, results of the same study indicate higher CKD incidence in patients with high sTfR, regardless of anemia and ID [95]. Although sTfR may be increased in patients undergoing HD and correlates inversely with iron storage, it is not possible to identify patients with iron deficiency during ESA therapy due to the increase in sTfR caused by increased erythropoiesis [96].

#### 3.5.2. Hepcidin

Hepcidin, a peptide hormone with an important role in iron homeostasis, contributes to ID and subsequent anemia formation. Hepcidin inhibits duodenal absorption of iron and release of iron from macrophages by ferroportin degradation, which is a major transporter of iron. There are several products of hepcidin N-terminal degradation such as hepcidin-24, -23, -22, and -20, whereas bioactive form is composed of 25 amino acids (hepcidin-25) [97]. According to impaired renal function, it has been shown that hepcidin-25 may be approximately 20-fold increased in patients with CKD when compared to healthy individuals [98]. Niihata et al. [99] investigated associations between hepcidin-25 and progression of renal anemia among non-dialyzed CKD patients. The results of this study indicate that higher levels of hepcidin-25 predict the progression of anemia in patients with sufficient iron stores. Moreover, in diabetic patients with CKD without ESA therapy, higher hepcidin-25 levels were associated with increased risk of CKD progression (HR 1.134 95% CI 1.041–1.235) and mortality (HR 1.49 95% CI 1.095–2.029) [99]. A study of Gao et al. [100] has shown that hepcidin-25 was negatively associated with Hb in CKD stage 5 patients, whereas there was no correlation between hepcidin-25 and reticulocyte hemoglobin equivalent. Negative correlation between hepcidin-25 and Hb has been also confirmed in a study of Niikura et al. [101] that assessed non-dialyzed, HD, and PD patients with CKD. The highest levels of hepcidin-25 were observed in patients undergoing PD [101]. To date, due to high variability and many cofounding factors (presented in table Y), hepcidin remains not useful for iron status assessment among CKD patients [102]. Negative correlation and predictive role in anemia progression suggest a potential role of hepcidin-25 in patients’ assessment; however, further studies are required to establish cutoffs; investigate its performance, sensitivity, and specificity; and compare it to other, highly applicable biomarkers.

#### 3.5.3. Neutrophil-Gelatinase-Associated Lipocalin

A few studies assessed the associations of neutrophil-gelatinase-associated lipocalin (NGAL) and ID in CKD patients. A correlation between functional ID (TSAT < 20%) anemia and NGAL serum concentration has been found, where NGAL has been correlated with serum ferritin and TIBC [103]. A study of Kim et al. [104] has revealed a correlation of NGAL with TSAT and serum ferritin. Moreover, it has been revealed that NGAL has better sensitivity and specificity in identifying iron deficiency than sTfR, transferrin, and ferritin [104,105]. Future studies are required to define cutoff values and a role of NGAL in ID evaluation. 

#### 3.5.4. Bone Metabolic Biomarkers

Chronic kidney disease bone and mineral disorders (CKD-BMD) are associated with poor prognosis due to bone abnormalities and extra-skeletal calcifications [106,107]. Based on CKD-BMD pathophysiology, several biomarkers such as FGF-23, klotho, phosphate, calcium, vitamin D, and PTH have been proposed to assess mineral status and estimate the risk for cardiovascular events, CKD to end-stage renal disease progression, and mortality [107]. 

A cohort study of Nam et al. [108] has shown that high FGF-23 levels were associated with lower Hb levels and increased risk of anemia development in non-dialyzed CKD patients. Moreover, FGF-23 correlated inversely with TSAT and serum iron, whereas positive correlation with hepcidin has been observed [108]. This phenomenon requires further studies to assess the role of FGF-23 in anemia formation; however, positive correlation between FGF-23 and hepcidin may suggest an indirect impact of FGF-23 on erythropoiesis. Thus, FGF-23 may have a role as a biomarker to identify patients at risk of anemia development. Moreover, recent studies assessed changes in FGF-23 levels in response to HIF-PH inhibitors, ESA, and iron supplementation. An animal model study of Clinckenbeard et al. [109] has shown that short-acting ESA administration was associated with increase in serum intact FGF-23. Interestingly, another study to assess the effect of ESA on FGF-23 serum concentrations revealed that long-acting ESA led to a decrease in intact FGF-23 and increase in C-terminal FGF-23 [110]. HIF-PH inhibitors are also speculated to enhance cleavage of intact FGF-23 and lead to its decrease and increase in C-terminal FGF-23 [111]. The presented studies indicate a potential role of FGF-23 not only in the risk assessment of anemia development but also in treatment monitoring. Further studies are required to determine the role of FGF-23 in anemia of CKD evaluation.

Biomarkers useful in anemia of CKD evaluation with factors influencing their values are presented in Table 3. 

## 4. Management

For the longest time, the primary treatment methods for anemia in CKD were erythropoiesis-stimulating agents (ESA) and oral or intravenous iron supplementation combined with the identification and management of anemia reversible causes. Reversible causes include vitamin B12/folate deficiency, hypothyroidism, and absolute iron deficiency. In severe cases, blood transfusions may also be necessary [57,116]. Although these therapies are well established, their efficiency remain variable, especially iron supplementation as its bioavailability may be decreased as a result of chronic inflammation and increased hepcidin levels, which in turn causes secondary anemia from ESA administration as it induces a high demand for iron that exceeds the supply [117,118].

### 4.1. Erythropoiesis-Stimulating Agents

ESAs come in a variety of different forms. Short-acting ESAs, such as epoetin α/β or epoetin κ, and long-acting ESAs, such as darbepoetin or epoetin β pegol, are commonly used in clinical practice [108]. The choice between short-acting and long-acting ESAs can impact patient outcomes. A study by Sakaguchi et al. [119] has shown higher mortality in patients receiving long-acting ESAs in comparison to short-acting ESAs. Yet, one key advantage of long-acting ESAs is their longer half-life, allowing for less frequent administration compared to short-acting ESAs like epoetin alfa and epoetin beta. This less frequent dosing regimen simplifies the work for healthcare providers in hemodialysis centers and contributes to better hemoglobin stability [120]. Interestingly, the efficacy of ESAs seems to not be affected by the type of ESA or the dosing interval, as observed in patients receiving chronic peritoneal dialysis [121].

Even though ESAs can effectively raise hemoglobin levels, there are concerns regarding their safety, including a potential increased risk of cancer progression and cardiovascular events with higher doses [122]. Neoplastic cells were shown to express erythropoietin receptors, which may constitute the underlying mechanism of possible cancer progression [123]. Another problem with ESAs is a phenomenon called “therapeutic inertia”, which refers to the lack of adequate iron or ESA prescriptions despite low hemoglobin levels and/or iron deficiency in patients with CKD [124]. This may lead to chaotic changes in hemoglobin levels and increase the risk of unfavorable outcomes, including renal death [125]. It is essential to overcome therapeutic inertia in order to guarantee prompt and suitable administration of ESAs and iron supplements. In fact, research conducted by Stauffer and Fan indicated that merely 22.8% of patients with chronic kidney disease and anemia had received treatment for their condition in the past three months [56]. Additionally, in the 1990s, researchers found that some patients continue to experience anemia even after taking high doses of ESAs, concluding that about 10% of patients are not responding well to this type of treatment [21]. More studies are needed to properly understand the nuances in different ESAs’ use and their impact on patient outcomes.

### 4.2. Hypoxia-Inducible Factor-Prolyl Hydroxylase Inhibitors

Hypoxia-inducible factor-prolyl hydroxylase inhibitors (HIF-PHI) have emerged as a novel class of therapeutic agents for the treatment of anemia in CKD. The mechanism of action of HIF-PHI involves the regulation of the HIF pathway [126]. The HIF-PHI mechanism of action is based on HIF stabilization, thereby it mimics the physiological response to hypoxia and promotes the transcription of genes involved in erythropoiesis, including EPO. This leads to increased EPO production and subsequent stimulation of erythropoiesis, addressing the underlying cause of anemia in CKD [127].

Roxadustat, a widely used HIF-PHI, has demonstrated clinical curative effects and safety in the management of anemia in CKD patients that either did not receive [128] or undergo dialysis [129]. Clinical trials have shown that roxadustat can significantly reduce hepcidin and may be a potential treatment for inflammation-induced anemia in CKD addressing ESA hyporesponsiveness [130,131]. Moreover, better efficacy in improving iron biomarker levels than ESA in real-world settings has been shown [132]. Similarly, other HIF-PHI, such as molidustat, daprodustat, vadadustat, and desidustat, have also shown efficacy in clinical trials, demonstrating the potential of the new drugs [133,134]. Recent studies suggest that roxodustat seems to be the best at improving hemoglobin levels when compared to other HIF-PHI [135]. The clinical efficiency of HIF-PHI has been further supported by their potential to replace ESA therapy and minimize the inconvenience to patients as HIF-PH can be taken orally in contrast to the intravenous or subcutaneous ESA [9]. In fact, daprodustat has been proven to be noninferior to ESA when administered just thrice weekly, during hemodialysis sessions, which improves patients’ adherence [136]. The results of the ASCEND-NHQ clinical trial indicate that daprodustat improves fatigue without an increase in the overall frequency of adverse events in comparison to placebo [137]. Recent meta-analysis also emphasized the lack of difference in cardiac and kidney adverse events in comparison to ESA and placebo [138].

In addition to promoting the production of EPO, HIF-PHI have been shown to have potential clinical benefits in enhancing iron mobilization and addressing the limitations of iron storage markers commonly used in clinical practice [16]. 

It should be noted that use of HIF-PHI is associated with certain significant concerns that must be considered. Studies indicate that activating HIF through these inhibitors could potentially boost the growth, spread, and metastatic potential of cells that have undergone malignant transformation, raising concerns about their safety in cancer patients [139]. Furthermore, the effects of HIF-PHI on heart health outcomes in anemic individuals with CKD when compared to ESAs are unclear and still a subject of ongoing research, with a hypothesis that these inhibitors may offer advantages in reducing cardiovascular risks, but more evidence is needed to properly assess this question [140].

Given the similar number of adverse events of HIF-PHI as ESA, with comparable efficacy, but with the convenience of the pill, HIF-PHI emerged as a small revolution with the multifaceted clinical implications in the management of anemia associated with CKD [141]. 

### 4.3. Anti-Bone Morphogenetic Protein 6 Antibodies

While HIF-PH inhibitors have shown promising effects in anemia of CKD, recent studies have also investigated the potential of anti-bone morphogenetic protein 6 (anti-BMP-6) antibodies as a novel therapeutic approach, which may offer an alternative strategy to modulate iron metabolism and erythropoiesis. Positive therapeutic effects of fully human anti-BMP-6 antibodies have been shown in rodent models of anemia of chronic disease [142]. The anti-BMP-6 antibody is a promising therapeutic option to reduce the need for EPO and improve iron metabolism, addressing key aspects of anemia in CKD [142].

Large-scale clinical trials are needed to assess the effectiveness of inhibiting BMP signaling for the treatment of CKD related anemia in humans.

### 4.4. Sodium-Glucose Transport Protein 2 Inhibitors

The therapeutic effect of sodium-glucose transport protein 2 inhibitors (SGLT-2i) on anemia in CKD has been a subject of interest in recent research. Although SGLT-2i have been primarily studied for their effects on glycemic control and cardiovascular outcomes in patients with diabetes, they have been shown to increase Hb in patients with kidney disease and/or heart failure [143,144,145]. More studies are needed to properly evaluate their role in the course of anemia as the mechanism of action and clinical efficiency remain poorly understood [143,144].

### 4.5. Novel Iron Therapies

In addition to novel drugs, the improved version of the standard iron supplementation are the subjects of extensive research. Two of the most promising candidates are ferric maltol and sucrosomial iron. Ferric maltol is an oral form of iron therapy and consists of a stable complex of ferric (Fe31) iron with maltol (3-hydroxy-2-methyl-4-pyrone), which is a naturally occurring sugar derivative [146]. The complex form prevents the formation of iron hydroxide polymers, which improves the bioavailability of the iron in comparison to standard iron salts [147]. The efficiency of this iron form in CKD has been proven in a phase 3, double-blind, randomized, placebo-controlled trial (AEGIS-CKD) [148]. On the other hand, sucrosomial iron is a presentation of ferric pyrophosphate covered by a phospholipid and sucrester membrane [149]. Sucrester promotes ferric ion transport across the intestinal epithelium as a vesicle-like structure independent of the divalent metal transporter 1 carrier [150]. A randomized controlled trial of 99 patients receiving either sucrosomial iron or intravenous iron gluconate showed that oral liposomal iron is a safe and efficacious alternative to intravenous iron gluconate to correct anemia in non-dialysis CKD [151], which is especially important because the current oral iron forms are inferior to intravenous iron administration [152]. These novel oral iron supplements offer potential advantages in terms of tolerability, iron absorption, and gastrointestinal effects, providing new possibilities for iron supplementation in CKD-related anemia [147].

### 4.6. Ziltivekimab

Ziltivekimab is a human IgG1k therapeutic monoclonal antibody targeting IL-6, designed with specific amino acid substitutions in the Fc domain to enhance its serum half-life. It has a unique mechanism of action that targets the inflammatory part of the anemia pathophysiology [153]. The results of phase 2 RESCUE randomized clinical trial suggest that anti-inflammatory therapy with ziltivekimab may indeed significantly improve hemoglobin levels and iron homeostasis markers in patients with stage 3–5 CKD [154]. Conversely, the ZEUS trial tries to verify whether ziltivekimab can reduce the incidence of cardiovascular events in patients with CKD. While the results are promising, more studies are still needed to properly assess its use in patients with anemia.

### 4.7. Unaddressed Questions

Although there are multiple promising therapies being currently researched, there are still some unaddressed questions. Firstly, the association between lower calcium levels and high phosphorus concentrations and anemia in CKD patients has been determined, yet the clinical significance remains uncertain, highlighting the need for further investigation into these associations [155]. Secondly, the role of eryptosis, or programmed cell death of red blood cells, in contributing to anemia in CKD patients is still uncertain, necessitating a deeper understanding of this mechanism and its implications for anemia management [120]. Lastly, there are the challenges in addressing anemia in frail elderly patients with CKD and multiple comorbidities, hospitalized patients, and kidney transplant recipients who may have reduced response to iron and ESA therapy [156].

## 5. Conclusions

Anemia of CKD constates a global challenge as it is associated with poor quality of life and systemic complications leading to death. In recent years, several advances in the pathophysiology of CKD-related anemia have been proposed. Several studies indicate a major role of decreased EPO secretion due to impaired renal reactivity, inflammation, uremic toxemia, and blood loss in anemia of CKD; however, there is still a need for further studies to investigate novel pathophysiological pathways that constitute a target for emerging therapies. 

Although several guidelines have been published, there are many inconsistencies between recommendations, such as undefined cutoffs for baseline measurements to diagnose anemia among CKD patients. In comparison to healthy individuals, patients suffering from CKD have different iron metabolism that decreases diagnostic performance of well-established biomarkers such as ferritin or TSAT. It should be emphasized that emerging biomarkers such as sTfR and NGAL require further studies to investigate their sensitivity and specificity, as well as to define appropriate thresholds. Moreover, already recommended and relatively old measurements such as %HRC and CHr should be considered in anemia diagnosis, as they seem to have better sensitivity and specificity when compared to ferritin and TSAT.

The management of anemia in CKD may be changing soon with the introduction of novel therapeutic strategies such as HIF-PH inhibitors and anti-BMP-6 antibodies. These innovative approaches have shown potential in fostering erythropoiesis, facilitating iron mobilization, and overcoming the challenges associated with conventional therapies. HIF-PH has shown efficacy and safety in several randomized clinical trials, while anti-BMP6 antibodies have shown promising results in pre-clinical animal trials. These multifaceted implications of HIF-PH inhibitors and the promising future presented by BMP-6 antibodies underline the dynamism in options available to manage CKD-induced anemia. In addition to novel pharmaceutics, the need for improved versions of iron supplementation have also spawned numerous research studies regarding innovative oral iron supplements, such as ferric maltol and sucrosomial iron, offering promising avenues for improving iron supplementation and addressing anemia in CKD patients. Recently, the role of sodium-glucose transport protein 2 inhibitors has also been emphasized, with a few papers showing its potential effects on improving hemoglobin levels. There is still a need for large-scale clinical trials to fully determine the efficacy, safety, and long-term outcomes of these new therapeutic strategies in the comprehensive management of CKD-anemia.

## Figures and Tables

**Table 1:** Hb concentration measurement frequency in CKD patients without anemia.

CKD Stage	Testing Frequency (at Least)
G3	Once per year
G4–G5 non-dialyzed	Twice per year
G4–G5 dialyzed	Every 3 months

CKD—chronic kidney disease; Hb—hemoglobin.

**Table 2:** Differences in measurements to evaluate anemia according to different guidelines.

Guideline	Hb *	TSAT **	Ferritin **	CHr **	%HRC **
KDIGO 2012 [54]	F: <12 g/dL M: <13 g/dL	<30% ***	<500 ng/mL ***	-	-
KDOQI 2006 4 [59]	F: <12 g/dL M: <13.5 g/dL	≤20%	≤100 ng/mL in PD and ND ≤200 ng/mL in HD	-	-
Renal Association 2017 [57]	Men and postmenopausal F: <13 g/dL Premenopausal F: <12 g/dL	≤20%	<100 ng/mL in PD and ND <200 ng/mL in HD	≤29 pg	≥6%
ERBP 2017 [60]	F: <12 g/dL M: <13.5 g/dL (in males >70 years <13.2 g/dL)	<20% in ND *** <25% in HD and PD *** <30% in patients on ESA ***	<100 ng/mL in ND *** <300 ng/mL in HD, PD *** <300 ng/mL in patients on ESA ***	-	-

* To diagnose anemia; ** to diagnose iron deficiency; *** to introduce iron therapy. F—female; M—male; Hb—hemoglobin; TSAT—transferrin saturation; CHr—reticulocyte hemoglobin count; %HRC—percentage of hypochromic red cells; ERBP—European Renal Best Practice; KDIGO—Kidney Disease: Improving Global Outcomes; KDOQI—Kidney Disease Outcomes Quality Initiative; HD—hemodialysis; ND—non-dialyzed; PD—peritoneal dialysis; ESA—erythropoiesis stimulating agents.

**Table 3:** Factors influencing values of measurements to assess iron state.

	Increase	Decrease
Serum ferritin [67]	Inflammation, autoimmune disorders, infectious disease CKD and dialysis Liver failure Hemochromatosis Cancers Hemophagocytic lymphohistiocytosis	Iron deficiency
TSAT [77,78,79]	As a result of TIBC decrease: CKD and dialysis Inflammation Iron overload Anemia of chronic disease Malnutrition Decreased liver function As a result of iron increase: Increase during morning hours High-iron diet	As a result of iron decrease: inflammation iron deficiency decrease during night hours low iron-diet As a result of TIBC increase: iron deficiency
CHr [84,112,113]	Iron supplementation	Iron deficiency
%HRC [113]	Iron deficiency (functional and total) Inflammation Enhanced hematopoiesis	Iron supplementation
sTFR [96,114]	Enhanced hematopoesis Iron deficiency Hyperinsulinemia Hemolytic anemia Hereditary spherocytosis, sickle-cell anemia, thalassemia	Anemia of chronic disease Hypertransfusion Chronic kidney disease Chemotherapy Exercises, weight loss, improved glucose tolerance
Hepcidin-25 [97]	RBC transfusions Iron supplementation Low iron stores CKD and dialysis Inflammation, infections	Enhanced erythropoiesis Anemia Chronic liver disease Alcohol Testosterone and estrogens
NGAL [115]	Sepsis Malignancy ATN-AKI, renal ischemia-reperfusion injury, renal tissue injury	

ATN-AKI—acute tubular necrosis acute kidney injury; TSAT—transferrin saturation; CHr—reticulocyte hemoglobin count; %HRC—percentage of hypochromic red cells.

## Data Availability

The data used in this article were sourced from the materials mentioned in the References section.
